# Comparing BD Affirm VPIII to Xpert Xpress MVP and BD MAX Vaginal Panel for the diagnosis of vaginal infections

**DOI:** 10.1128/spectrum.02353-25

**Published:** 2026-07-28

**Authors:** Lisa A. Cosentino, Melinda Petrina, Tracy Zamborsky, May Beamer, Ingrid Macio, Leslie A. Meyn, Katherine E. Bunge, Sharon L. Hillier

**Affiliations:** 1 Magee-Womens Research Institute 132197, Pittsburgh, Pennsylvania, USA; 2 Department of Obstetrics, Gynecology and Reproductive Sciences, University of Pittsburgh 204912, Pittsburgh, Pennsylvania, USA; Houston Methodist Hospital, Houston, Texas, USA

**Keywords:** bacterial vaginosis, *Candida*, *Trichomonas vaginalis*

## Abstract

BD Affirm VPIII, a non-amplified molecular test for the detection of *Candida, Trichomonas vaginalis* (TV), and *Gardnerella vaginalis* (GV), was compared to two nucleic acid amplification tests (NAATs), the Xpert Xpress MVP and BD MAX Vaginal Panel, to estimate its sensitivity and specificity for the diagnosis of vaginitis/vaginosis. Women ≥14 years old, seeking care with symptoms of vaginitis (vaginal discharge, odor, vulvar or vaginal itch, irritation, and burning), provided self-collected vaginal samples for the Xpert Swab Specimen Collection (Xpert Xpress MVP), BD Molecular Swab Collection (BD MAX Vaginal Panel), and BD Affirm Ambient Temperature Transport System (BD Affirm VPIII). True positive and negative results were defined as concordant results on both NAATs. Agreement between the NAATs was 96% for bacterial vaginosis (BV), 95% for *Candida,* and 100% for TV. Based on both NAATs, the prevalence of BV in the population was estimated to be 42.8% (118/276), 28.3% (78/276) for *Candida,* and 5.1% (14/276) for TV. The BD Affirm VPIII detected only 43% of TV and 56% of *Candida.* While the sensitivity of GV for BV was 89.7% (95% confidence interval [CI]: 82.6–94.5), the specificity was 76.2% (95% CI: 68.4–82.9). Of women with no BV, *Candida*, or TV by NAAT, 29.5% would have been misdiagnosed based on Affirm VPIII. Reliance on GV for the diagnosis of BV results in false positives in women without BV and could trigger unwarranted treatment. The inadequate sensitivity of Affirm VPIII for *Candida* and TV and poor specificity for BV limits its utility in clinical settings for women with symptomatic vaginitis.

**IMPORTANCE:**

Vaginal infections are common among women of reproductive age, with substantial costs for women and health systems. Point-of-care methods widely used in clinical practice to diagnose bacterial vaginosis, vulvovaginal candidiasis, and trichomoniasis include microscopy, pH testing, and amine odor. Microscopy for motile trichomonads or the pseudohyphae of *Candida* is insensitive because *Candida* or *Trichomonas vaginalis* can cause symptoms even when present in concentrations not detectable by direct microscopy. When microscopy is not available, the U.S. Centers for Disease Control and Prevention has recommended the use of other tests, including nonamplified DNA probe technology. Our study compared this system to two FDA-cleared amplified testing systems for the diagnosis of vaginitis. The nonamplified system was insensitive for the detection of *Candida* and *Trichomonas vaginalis* and nonspecific for bacterial vaginosis, which could result in inappropriate treatment and misdiagnosis, limiting its clinical utility for use in women with vaginitis symptoms.

## INTRODUCTION

Bacterial vaginosis (BV), *Trichomonas vaginalis* (TV), and vulvovaginal candidiasis are common among reproductive-age women, accounting for an estimated 10 million office visits annually in the United States (1). BV and TV are associated with increased risk of sexually transmitted infections, including HIV (2, 3), pelvic inflammatory disease (4, 5), and preterm delivery (6, 7). The clinical burden of patients presenting with vaginal symptoms in the United States is substantial, and approximately 30% of women treated for vaginitis symptoms seek subsequent vaginitis care in the following 12 months (8). In a study of 140,826 commercially insured patients, 36% had BV recurrence over a mean of 28 months at an annual cost of $403 per year among those with one treatment course and $806 for those having four or more infections (9). Recurrent vaginal infections due to *Candida* also have substantial costs to health systems (10).

The Centers for Disease Control and Prevention (CDC) guidelines describe the point-of-care (POC) tests that can be performed as an adjunct to the clinical history and physical examination to support the diagnosis of vaginal discharge syndromes (11). These tests include measurement of vaginal pH, “whiff” test (addition of potassium hydroxide [KOH] to vaginal fluid for the assessment of amine odor), and wet mount slide for microscopic examination of fresh samples of the discharge to identify the presence of clue cells, motile trichomonads, and/or budding yeast/pseudohyphae. The sensitivity and specificity of microscopic detection of clue cells, yeast, and trichomonads by clinicians vary considerably (12–14), and pH and amine odor testing are infrequently performed due to lack of access to these simple tests (15).

The BD Affirm VPIII Microbial Identification test (Affirm VPIII) (Becton Dickinson) is a DNA probe test intended for use in the detection and identification of *Candida*, *Gardnerella vaginalis* (GV), and TV. It is a moderately complex test that can provide results in less than an hour in clinical care settings with a CLIA-licensed laboratory. It is the most commonly used lab-based test, accounting for 20% of diagnostic testing performed in one retrospective analysis of nearly 4 million women seeking care for vaginitis in the United States (8). In a study conducted in community practice settings, more than 75% of vaginitis visits did not include wet mount, pH testing, or whiff test (16), but 73% of clinician visits for vaginitis included testing using Affirm VPIII. The Affirm VPIII test does not identify *Candida glabrata* or *Candida krusei* separately but detects them as part of a single *Candida* target. It also detects GV as a marker indicative of BV; however, GV can also be detected in women having a *Lactobacillus*-dominated vaginal microbiome and does not always indicate BV (17). This lack of specificity underlies the risk of false-positive results and misdiagnosis. As a mitigation strategy, the use of pH and amine odor testing has been recommended to improve the specificity of the Affirm GV test (18), and the use of these two POC tests is recommended by the CDC as an adjunct to Affirm VPIII testing (11).

FDA-cleared nucleic acid amplification tests (NAATs) for the diagnosis of vaginitis include the BD MAX Vaginal Panel (BD MAX VP) (19) and the Hologic Aptima *Candida/Trichomonas* Vaginitis assay (20), which have high throughputs but rely on equipment that is not available at the POC. The Xpert Xpress MVP (Xpress MVP) is a CLIA-waived, FDA-cleared on-demand NAAT that can be performed by untrained operators (21) to aid in the detection of vaginal infections in women with a clinical presentation of vaginitis/vaginosis.

Given the limitations of the non-amplified DNA probe system (22–26) and the availability of a CLIA-waived NAAT for vaginal infections, our objective was to evaluate the performance of the Affirm VPIII test in comparison to the results of two NAAT platforms for the detection of vaginitis/vaginosis: Xpress MVP and BD MAX VP. A second objective was to perform new statistical analyses of published studies of the Affirm VPIII to provide a consolidated assessment of the sensitivity and specificity of the Affirm VPIII against an array of comparators.

## MATERIALS AND METHODS

### Study participants

The study was performed from 3 July 2024 to 31 March 2025. Women 14 years and older, having symptoms of vaginitis (vaginal discharge, vaginal odor, vulvar or vaginal itch, vulvar/vaginal irritation, burning, pain, and/or vulvar edema), and seeking care at 7 OB-Gyn clinics and 90 providers within a single medical center were approached.

### Sample collection

Women provided self-collected vaginal samples with the Xpert Swab Specimen Collection Kit for the Xpress MVP (Cepheid, Sunnyvale, CA, USA), the BD Molecular Swab Collection for the BD MAX VP, and the BD Affirm Ambient Temperature Transport System (ATTS) for the Affirm VPIII (Becton Dickinson, Sparks, MD, USA). The order of the swab collection was randomized to prevent sampling bias.

### Sample testing

Upon collection, the vaginal swab for Affirm VPIII was placed in the ATTS transport tube and sent to the lab for testing. Affirm VPIII testing was performed and interpreted according to the manufacturer’s instructions. The Xpert Swab Specimen Collection kit was either tested immediately in the clinic or transported to the lab for testing to be performed within 14 days. The Xpress MVP test was performed on the GeneXpert Xpress System, an automated qualitative *in vitro* diagnostic test for the detection of DNA targets from anaerobic bacteria associated with bacterial vaginosis, namely *Atopobium vaginae* (now *Fannyhessea vaginae*) (27), bacterial vaginosis-associated bacteria 2 (now *Amygdalobacter indicium*) (28), and *Megasphaera-*1 (now *Megasphaera lornae*) (29), *Candida* group (*Candida albicans*, *Candida tropicalis*, *Candida parapsilosis*, and *Candida dublinensis*) and *C. glabrata*/*C. krusei*-associated with vulvovaginal candidiasis, and TV. The GeneXpert Xpress software interprets the measured fluorescent signals and embedded calculation algorithms based on the presence and relative abundance of the targets and provides test results for BV, *Candida* group, *Candida glabrata-krusei,* and TV.

The BD Molecular Swab Collection tube was transported to the lab and tested using the BD MAX VP, which is an assay that utilizes fluorogenic target-specific probes for the qualitative identification of BV-specific organisms, including *G. vaginalis*, *Atopobium vaginae*, bacterial vaginosis-associated bacteria 2, *Megasphaera-*1, and *Lactobacillus* species (*Lactobacillus crispatus* or *Lactobacillus jensenii*); the *Candida* group (*C. albicans*, *C. tropicalis*, *C. parapsilosis*, and *C. dublinensis* species), *C. glabrata,* and *C. krusei*; and TV, and assigns a positive or negative result based on the relative concentrations of organisms present. The panel runs on the BD MAX System, an automated platform that combines PCR extraction, amplification, and detection. Test results are provided for BV, *Candida*, *C. glabrata*, *C. krusei*, and TV.

### Analyses

True positives were defined as samples positive by both FDA-cleared amplified testing systems. Sensitivity and specificity are presented along with corresponding exact binomial 95% confidence intervals (CIs). The frequency of diagnoses between the testing systems was compared using the binomial test of proportions. For the comparison of Affirm VPIII sensitivity and specificity to other test systems in Table 3, confidence intervals were recalculated using the exact binomial method for consistency across multiple studies.

## RESULTS

We enrolled 276 women with a median age of 33 years (range, 17–76); 49.6% self-reported as black, 40.6% as white, and 9.8% as other. Twenty (7.2%) participants were pregnant. Participants reported that previous vaginal infections were common, with 70% of study participants reporting one or more vaginal infections in the previous 12 months. Recurrent vaginitis, defined as four or more infections in the past 12 months, was reported by 53 (19.2%) of the study participants. The prevalence of vaginal infections detected by the two FDA-cleared NAATs and the Affirm VPIII is shown in Table 1. The Xpress MVP and the BD MAX VP had similar frequencies of detection for BV, *Candida,* and TV. The Affirm VPIII identified more women as BV-positive based on the detection of GV compared to the two molecular tests for BV and only about half of the *Candida* and TV detected by the NAATs. Of the 276 Xpress MVP tests, 139 Xpress MVP tests were performed by clinicians and 137 by laboratory personnel. All BD MAX VP were tested in the laboratory. Of the 32 discordant results between BD MAX VP and Xpress MVP, 18 Xpress MVP tests were performed in the laboratory, and 14 by clinic staff.

**TABLE 1:** Prevalence of vaginal infections detected by Xpress MVP, BD MAX VP, and BD Affirm VPIII in 276 women presenting with symptoms^*h*^

Detection	Xpress MVP	BD MAX VP	Concordant^*a*^	BD Affirm VPIII^*b*^	*P* value^*c*^	*P* value^*d*^	*P* value^*e*^
Bacterial vaginosis^*f*^	122 (44.2%)	126 (45.7%)	118 (42.8%)	146 (53.9%)	0.023	0.055	0.009
*Candida* species^*g*^	87 (31.5%)	84 (30.4%)	78 (28.3%)	46 (17.0%)	<0.001	<0.001	0.002
*C. glabrata/krusei*	11 (4.0%)	12 (4.3%)	8 (2.9%)	NA	NA	NA	NA
*Trichomonas vaginalis*	14 (5.1%)	14 (5.1%)	14 (5.1%)	6 (2.2%)	0.071	0.071	0.071

^*a*^ Concordant result for Xpress MVP and BD MAX VP.

^*b*^ Percentages are out of 271 since 5 tests were invalid.

^*c*^ Xpress MVP vs BD Affirm VPIII.

^*d*^ BD MAX VP vs BD Affirm VPIII.

^*e*^ Concordant NAATs vs BD Affirm VPIII.

^*f*^ *Gardnerella vaginalis* for the BD Affirm VPIII test.

^*g*^ *Candida albicans, C. tropicalis, C. dubliniensis, *and* C. parapsilosis.*

^*h*^ NA, not applicatcable.

**Fig 1:**
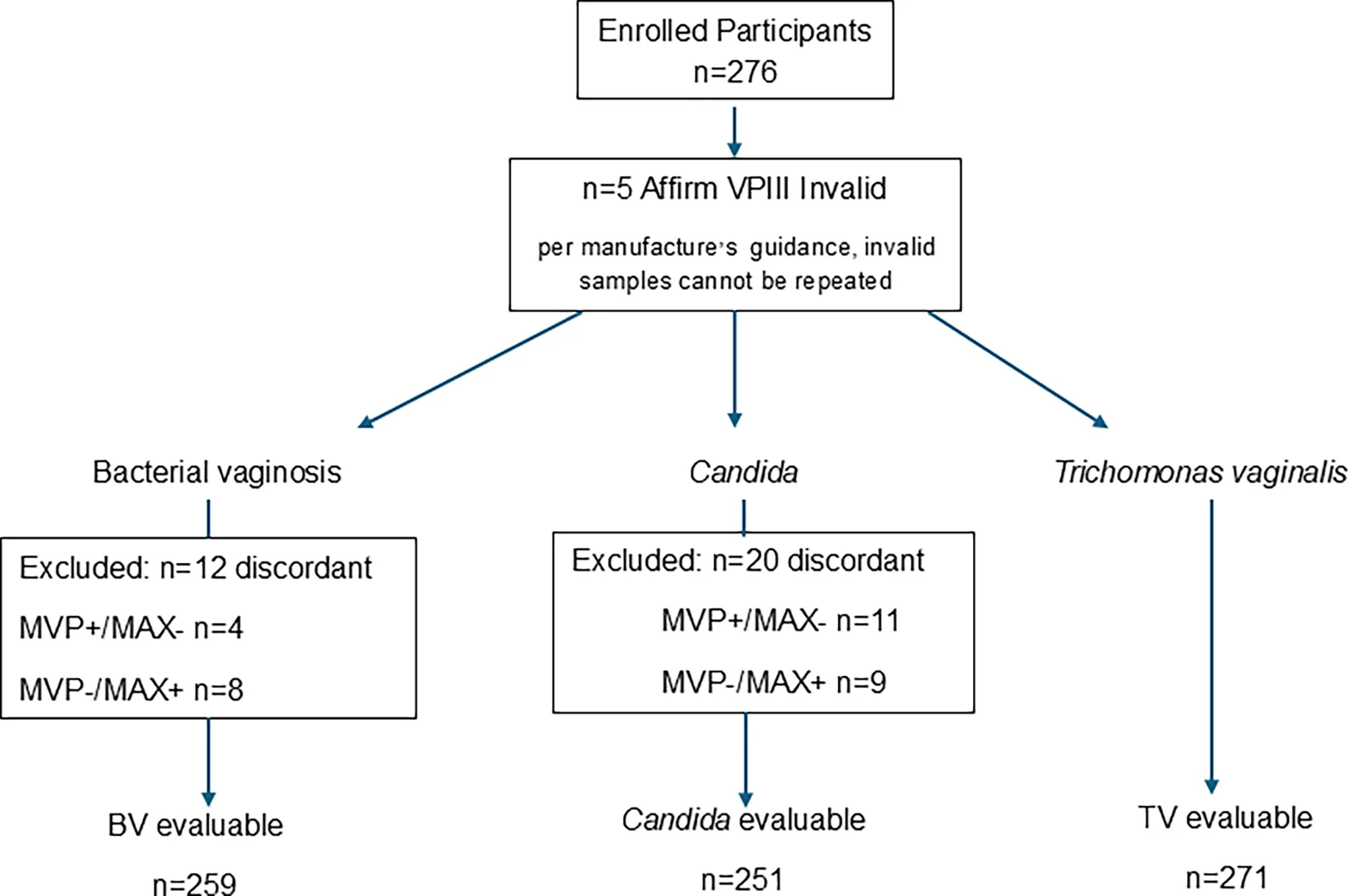
Evaluable study population for each test based on concordant NAAT results. Reasons for exclusion included invalid Affirm VPIII results or discordant results between the Xpert Xpress MVP (MVP) and BD MAX VP (MAX) tests.

The evaluable study population for each test based on concordant NAAT results is shown in Fig. 1. Five samples were excluded from the analysis because of invalid Affirm VPIII test results due to an instrument error. In total, 20 *Candida* and 12 BV samples were excluded from the analysis due to discordant results between the two NAAT assays, leaving an analysis population of 259 for BV, 251 for *Candida,* and 271 for TV.

The distribution of vaginal infections as diagnosed by the two concordant FDA-cleared NAATs vs the Affirm VPIII test is shown in Table 2 for the 239 women having two concordant NAATs for all three conditions. Although women in the study were not treated based on the Affirm results, if the diagnosis had relied solely on the Affirm VPIII test without pH and amine odor testing, 8.3% of women with BV, 48.8% of women with *Candida,* and 100% of women with TV would have been misdiagnosed. Of the 239 women, 34 (14%) had more than one condition detected by NAAT, and misdiagnosis based on Affirm alone was 20/34 (58.8%). Of women having no NAAT-confirmed condition, 29.5% would have been diagnosed inappropriately based on Affirm testing alone. Overall, 27.2% of women would have been misdiagnosed, leading to potential inappropriate treatment. Of these, 21 women having symptoms of vaginitis and *Candida* detected by both NAATs would have been informed that no pathogen was detected, and 26 women with no condition detected based on two NAATs would have been misdiagnosed as having BV. The 34 women having more than one diagnosed condition by NAAT could have received treatment for only one condition when two were present. Of the 19 pregnant people in the study having two concordant NAATs, 4 had both BV and *Candida*, 6 had BV alone, 6 had *Candida* alone, and 3 had no diagnosed condition. Affirm results were available for 18 of these participants, and in this subset, 7/18 (39%) would have been incorrectly diagnosed based on Affirm alone, similar to the overall population (27%).

**TABLE 2:** Distribution of vaginal infections as diagnosed by two concordant FDA-cleared NAATs and Affirm VPIII test positivity and proportion, which would have been misdiagnosed if based on Affirm VPIII without pH and KOH testing

Detection by Xpress MVP and BD MAX	*N* (%)	BD Affirm VPIII positive			No. (%) misdiagnosed based on BD Affirm VPIII
		*G. vaginalis*	*Candida*	*T. vaginalis*	
Bacterial vaginosis	72	66 (91.7%)	0	0	6 (8.3%)
*Candida*	43	8 (18.6%)	27 (62.8%)	0	21 (48.8%)^*a*^
*Trichomonas vaginalis*	2	0	0	0	2 (100%)
BV and TV	6	5 (83.3%)	0	4 (66.7%)	3 (50%)
BV and *Candida*	26	23 (88.5%)	12 (46.2%)	0	15 (57.7%)^*b*^
*Candida* and TV	1	0	0	0	1 (100%)
BV, *Candida*, and TV	1	1 (100%)	0	0	1 (100%)
No condition detected	88	26 (29.5%)	1 (1.1%)	0	26 (29.5%)^*c*^
Total	239	129 (54.0%)	40 (16.7%)	4 (1.7%)	65 (27.2%)

^*a*^ A total of 3 women were misdiagnosed as having BV when none was present, 5 with BV and *Candida* when no BV was present, and 13 were diagnosed as having no pathogen when one or more were detected by NAAT.

^*b*^ A total of twelve women were misdiagnosed as having BV alone when both BV and *Candida* were present, 1 woman was diagnosed as *Candida* alone when both BV and *Candida* were present, and 2 women with no vaginal condition were misdiagnosed as having BV based on *G. vaginalis*.

^*c*^ A total of 25 women were diagnosed as having BV based on the detection of *G. vaginalis* when BV was not detected by NAAT, and 1 woman was diagnosed as having *Candida *and *G. vaginalis* by Affirm when neither BV nor *Candida* was detected by NAAT.

The sensitivity and specificity of the Affirm VPIII system in our study were compared to other studies enrolling different populations of women and using various comparators (Table 3). The sensitivity and specificity of the Affirm VPIII in the present study were 104/116 (89.7%, 95% confidence interval: 82.6%–94.5%) and 109/143 (76.2%, 95% CI: 68.4%–82.9%) for GV, 45/80 (56.3%, 95% CI: 44.7%–67.3%) and 170/171 (99.4%, 95% CI: 96.8%–100%) for *Candida,* and 6/14 (42.9%, 95% CI: 17.7%–71.1%) and 257/257 (100%, 95% CI: 98.6%–100%) for TV (Table 3). All the published studies had similar sensitivity and specificity to those of the present study for BV, *Candida,* and TV.

**TABLE 3:** Summary of the reported sensitivity and specificity of the Affirm VPIII in the present study and how it compares to other published studies

Condition/study	Number of participants	Comparator/molecular diagnostic method	BD Affirm VPIII assay	
			Sensitivity % (95% CI)	Specificity % (95% CI)
Bacterial vaginosis				
Cosentino et al. (current study)	259	Xpert Xpress MVP and BD MAX Vaginal Panel	89.7 (82.6–94.5)	76.2 (68.4–82.9)
Cartwright et al. (22)	305	Lab-developed PCR *Atopobium, Megasphaera-1*, BVAB-2, *G. vaginalis* (BV-PCR)	90.9 (85.9–94.5)^*a*^	67.6 (57.9–76.3)^*a*^
Thompson et al. (23)	182	BD MAX Vaginal Panel	96.2 (89.3–99.2)	81.6 (72.7–88.5)
Richter et al. (24)	111	Hologic ASR BV/Aptima IVD BV assay	86.7 (73.2–94.9)^*a*^	60.6 (47.8–72.4)^*a*^
Dessai et al. (25)	251	BD MAX Vaginal Panel	79.8 (71.7–86.5)	80.3 (72.3–86.8)
Summary statistic for BV	1,108		88.6 (85.7–91.1)	74.6 (70.7–78.2)
*Candida*				
Cosentino et al. (current study)	251	Xpert Xpress MVP and BD MAX Vaginal Panel	56.3 (44.7–67.3)	99.4 (96.8–100.0)
Cartwright et al. (22)	102	Lab-developed *Candida* PCR	58.1 (42.1–73.0)^*a*^	100.0 (93.9–100.0)^*a*^
Thompson et al. (23)	193	BD MAX Vaginal Panel	69.4 (56.3–80.4)	100.0 (97.2–100.0)
Richter et al. (24)	111	Aptima ASR *Candida* assay	75.9 (56.5–89.7)^*a*^	98.8 (93.4–100.0)^*a*^
Dessai et al. (25)	271	BD MAX Vaginal Panel	52.9 (44.7–61.0)	97.4 (92.6–99.5)
Summary statistic for *Candida*	928		58.8 (53.6–63.9)	99.1 (97.9–99.7)
*Trichomonas vaginalis*				
Cosentino et al. (current study)	271	Xpert Xpress MVP and BD MAX Vaginal Panel	42.9 (17.7–71.1)	100.0 (98.6–100.0)
Cartwright et al. (22)	320	Gen-Probe Aptima TV	46.3 (32.6–60.4)^*a*^	100.0 (98.6–100.0)^*a*^
Thompson et al. (23)	192	BD MAX Vaginal Panel	100.0 (63.1–100.0)^*a*^	100.0 (98.0–100.0)^*a*^
Dessai et al. (25)	271	BD MAX Vaginal Panel	46.4 (27.5–66.1)	100.0 (98.5–100.0)
Andrea et al. (26)	781	Gen-Probe Aptima TV	63.4 (46.9–77.9)^*a*^	99.9 (99.2–100.0)^*a*^
Summary statistic for TV	1,835		53.8 (45.3–62.1)	99.9 (99.7–100.0)

^*a*^ Confidence intervals recalculated using exact binomial method for consistency across published manuscripts.

## DISCUSSION

In this study of 276 women seeking care for vaginitis symptoms at hospital-based primary care clinics, the Affirm VPIII had low sensitivity for detecting *Candida* and TV and moderate specificity for detecting BV. Other studies that have compared the Affirm VPIII results to various molecular diagnostic methods for diagnosis of vaginal infections have also reported low sensitivity of this system for detecting *Candida* and TV and moderate specificity for detecting BV (22–26). Although this nonamplified probe test system represented a technological breakthrough in the mid-1990s, the availability of NAATs, which have greater sensitivity and specificity for diagnosis of vaginal infections, has limited the clinical utility of the nonamplified probe test today.

The low/moderate specificity of Affirm VPIII for BV detection is likely due to its reliance on detection of GV*,* which is a nonspecific indicator of BV (17). The sensitivity of Affirm VPIII in the five studies included in Table 3 ranged from 79.8% to 96.2%, while the specificity for BV was 60.6%–81.6%. Affirm VPIII relies on GV detection alone, which is a nonspecific marker also found in women with *Lactobacillus*-dominated microbiota. The CDC recommends that providers use Affirm in conjunction with vaginal pH testing and KOH testing for amine odor (11) to increase the specificity of BV diagnosis. However, in our study, including 90 providers in a setting with pH paper and KOH available, only 23.6% of patient records reported a pH paper result and 44.0% reported amine odor testing. Nyirjesy et al. (15) reported that 34% of providers reported a lack of access to KOH and 41% reported a lack of access to pH paper in their clinics.

The Affirm VPIII test had lower sensitivity for detection of *Candida* than NAATs, ranging from 52.9% to 75.9%, while the specificity was high, 97.4%–100% across multiple studies. For *Candida*, the Xpress MVP and BD MAX VP detect several species (*C. albicans, C. tropicalis, C. parapsilosis*, and *C. dubliniensis*). The assays differ in that *C. glabrata/C. krusei* is grouped for Xpress MVP, whereas they are separate targets for BD MAX VP. The Hologic Aptima Candida/Trichomonas vaginitis assay detects *Candida* (*C. albicans, C. tropicalis, C. parapsilosis*, and *C. dubliniensis*) and *C. glabrata* (20). The less frequently detected *C. glabrata and C. krusei* are relevant because they are known to be more difficult to treat with azole therapy (30), but reliance on the nonamplified probe system does not provide this information, which can be used to guide appropriate therapy.

TV is sexually transmitted, and its detection should trigger treatment of women and their sexual partners to prevent reinfection. TV was infrequent in our study population of women with vaginitis attending obstetrics and gynecology clinics, limiting the statistical power of our study to differentiate the performance of Affirm VPIII from the two NAATs. In the current study, Affirm VPIII failed to detect 8 (57%) of 14 TV cases. In a published study that included 41 TV infections, the sensitivity of Affirm VPIII was 63% (95% CI: 55%–65%), which suggests that our estimated sensitivity was similar to a study with greater statistical power (26). Thompson et al. (23) reported 100% concordance between Affirm VPIII and NAATs for the detection of TV, but the authors attributed the high level of sensitivity to the small number of TV infections. TV is often a component of a mixed vaginal infection. Hillier reported that 63% of women having TV had mixed infections (16). In a study by Andrea and Chapin (26), 6 of 10 cases of TV were missed by Affirm VPIII, with 4 of the 6 having BV and TV. Therefore, women misdiagnosed by Affirm as having only BV would have received BV treatment (oral metronidazole), which may incidentally treat TV, but their sexual partners would not have been appropriately treated for TV. Given the availability of more sensitive NAATs, reliance on Affirm VPIII for diagnosing TV is not appropriate and could lead to missed infections.

An advantage of the Xpress MVP test is that it yields results at the POC by clinical staff, which are similar to those when the testing is performed by laboratory personnel (21), and this was confirmed in our study. Because Xpress MVP can generate results in less than an hour, requires minimal (less than 5 min) setup, and is user-friendly, it is suitable for use as a POC test. The Affirm VPIII, while easy to perform, requires more attention and processing time compared to the Xpress MVP and is reliant on a colorimetric change on the bead, which may be subjective.

Our study has several strengths and important limitations. Our study randomized the order of swab collection to minimize bias and included women from multiple clinics and 90 different providers, providing a real-world evaluation of BD Affirm VPIII test performance. Another strength was the use of two FDA-cleared testing systems to define true positives. A potential limitation of using two testing systems to define true positives was that, while this approach ensures a highly specific comparator, it may bias sensitivity and specificity estimates by removing more challenging or ambiguous cases. To exclude this possibility, the sensitivity and specificity of the Affirm test were evaluated using any FDA-cleared NAAT positives with nearly identical results (Table S1). A limitation of our study was the lack of Nugent score, which would have been useful to resolve discordant BV results. A yeast culture could have identified which *Candida* species were present in samples from women having discordant NAATs. Although the BD Affirm VPIII test was not validated for use with self-collected samples in our study, the level of agreement with the FDA-cleared tests was similar to the published performance of Affirm VPIII using clinician-collected swabs.

Women who seek care for vaginitis deserve an accurate diagnosis, which can lead to the provision of appropriate treatment. Incorrect diagnosis of vaginal infections and inappropriate treatment impose costs on the people seeking care, their providers, and health systems. Inappropriate use of antimicrobials can contribute to antimicrobial resistance, disrupt the vaginal microbiome, and cause side effects. The results of the present study suggest that the Affirm VPIII non-amplified testing system lacks sensitivity and specificity and has limited clinical utility for use in primary care settings for the diagnosis of vaginal infections.

## Data Availability

The data from this study are available on request from the authors and will include deidentified data and performance data for each testing system used.

## SUPPLEMENTAL MATERIAL

Table S1 spectrum.02353-25-s0001.docx Sensitivity and Specificity of Affirm VPIII for Bacterial Vaginosis (BV), *Candida* species, and *Trichomonas vaginalis*.

ASM does not own the copyrights to Supplemental Material that may be linked to, or accessed through, an article. The authors have granted ASM a non-exclusive, world-wide license to publish the Supplemental Material files. Please contact the corresponding author directly for reuse.

## References

[1] Brown H, Drexler M. 2020. Improving the diagnosis of vulvovaginitis: perspectives to align practice, guidelines, and awareness. Popul Health Manag 23:S3–S12. doi:10.1089/pop.2020.0265 32997581 PMC7591372

[2] Atashili J, Poole C, Ndumbe PM, Adimora AA, Smith JS. 2008. Bacterial vaginosis and HIV acquisition: a meta-analysis of published studies. AIDS 22:1493–1501. doi:10.1097/QAD.0b013e3283021a37 18614873 PMC2788489

[3] Kissinger P, Adamski A. 2013. Trichomoniasis and HIV interactions: a review. Sex Transm Infect 89:426–433. doi:10.1136/sextrans-2012-051005 23605851 PMC3748151

[4] Ravel J, Moreno I, Simón C. 2021. Bacterial vaginosis and its association with infertility, endometritis, and pelvic inflammatory disease. Am J Obstet Gynecol 224:251–257. doi:10.1016/j.ajog.2020.10.019 33091407

[5] Cherpes TL, Wiesenfeld HC, Melan MA, Kant JA, Cosentino LA, Meyn LA, Hillier SL. 2006. The associations between pelvic inflammatory disease, *Trichomonas vaginalis* infection, and positive herpes simplex virus type 2 serology. Sex Transm Dis 33:747–752. doi:10.1097/01.olq.0000218869.52753.c7 16691155

[6] Mohanty T, Doke PP, Khuroo SR. 2023. Effect of bacterial vaginosis on preterm birth: a meta-analysis. Arch Gynecol Obstet 308:1247–1255. doi:10.1007/s00404-022-06817-5 36251068

[7] Silver BJ, Guy RJ, Kaldor JM, Jamil MS, Rumbold AR. 2014. *Trichomonas vaginalis* as a cause of perinatal morbidity: a systematic review and meta-analysis. Sex Transm Dis 41:369–376. doi:10.1097/OLQ.0000000000000134 24825333

[8] Chen J, Tse J, Shi L, Cheng MM, Lillis R, Near AM. 2025. Real-world clinical burden of patients presenting with vaginitis symptoms in the United States. AJOG Glob Rep 5:100504. doi:10.1016/j.xagr.2025.100504 40524705 PMC12167788

[9] Watkins E, Chow CM, Lingohr-Smith M, Lin J, Yong C, Tangirala K, Collins K, Li J, Brooks R, Amico J. 2024. Treatment patterns and economic burden of bacterial vaginosis among commercially insured women in the USA. J Comp Eff Res 13:e230079. doi:10.57264/cer-2023-0079 38099520 PMC10842271

[10] Denning DW, Kneale M, Sobel JD, Rautemaa-Richardson R. 2018. Global burden of recurrent vulvovaginal candidiasis: a systematic review. Lancet Infect Dis 18:e339–e347. doi:10.1016/S1473-3099(18)30103-8 30078662

[11] Workowski KA, Bachmann LH, Chan PA, Johnston CM, Muzny CA, Park I, Reno H, Zenilman JM, Bolan GA. 2021. Sexually transmitted infections treatment guidelines, 2021. MMWR Recomm Rep 70:1–187. doi:10.15585/mmwr.rr7004a1 PMC8344968 34292926

[12] Bornstein J, Lakovsky Y, Lavi I, Bar-Am A, Abramovici H. 2001. The classic approach to diagnosis of vulvovaginitis: a critical analysis. Infect Dis Obstet Gynecol 9:105–111. doi:10.1155/S1064744901000187 11495550 PMC1784643

[13] Nathan B, Appiah J, Saunders P, Heron D, Nichols T, Brum R, Alexander S, Baraitser P, Ison C. 2015. Microscopy outperformed in a comparison of five methods for detecting *Trichomonas vaginalis* in symptomatic women. Int J STD AIDS 26:251–256. doi:10.1177/0956462414534833 24855131

[14] Wendel KA, Erbelding EJ, Gaydos CA, Rompalo AM. 2002. *Trichomonas vaginalis* polymerase chain reaction compared with standard diagnostic and therapeutic protocols for detection and treatment of vaginal trichomoniasis. Clin Infect Dis 35:576–580. doi:10.1086/342060 12173132

[15] Nyirjesy P, Banker WM, Bonus TM. 2020. Physician awareness and adherence to clinical practice guidelines in the diagnosis of vaginitis patients: a retrospective chart review. Popul Health Manag 23:S13–S21. doi:10.1089/pop.2020.0258 32985960 PMC7591374

[16] Hillier SL, Austin M, Macio I, Meyn LA, Badway D, Beigi R. 2021. Diagnosis and treatment of vaginal discharge syndromes in community practice settings. Clin Infect Dis 72:1538–1543. doi:10.1093/cid/ciaa260 32350529 PMC8248297

[17] Srinivasan S, Fredricks DN. 2008. The human vaginal bacterial biota and bacterial vaginosis. Interdiscip Perspect Infect Dis 2008:750479. doi:10.1155/2008/750479 19282975 PMC2648628

[18] Briselden AM, Hillier SL. 1994. Evaluation of affirm VP microbial identification test for *Gardnerella vaginalis* and *Trichomonas vaginalis*. J Clin Microbiol 32:148–152. doi:10.1128/jcm.32.1.148-152.1994 8126171 PMC262986

[19] Schwebke JR, Gaydos CA, Nyirjesy P, Paradis S, Kodsi S, Cooper CK. 2018. Diagnostic performance of a molecular test versus clinician assessment of vaginitis. J Clin Microbiol 56:e00252–18. doi:10.1128/JCM.00252-18 29643195 PMC5971525

[20] Schwebke JR, Taylor SN, Ackerman R, Schlaberg R, Quigley NB, Gaydos CA, Chavoustie SE, Nyirjesy P, Remillard CV, Estes P, et al.. 2020. Clinical validation of the Aptima bacterial vaginosis and Aptima *Candida/Trichomonas vaginitis* assays: results from a prospective multicenter clinical study. J Clin Microbiol 58:e01643-19. doi:10.1128/JCM.01643-19 31748322 PMC6989072

[21] Lillis RA, Parker RL, Ackerman R, Ackerman J, Young S, Weissfeld A, Trevino E, Nachamkin I, Crane L, Brown J, et al.. 2023. Clinical evaluation of a new molecular test for the detection of organisms causing vaginitis and vaginosis. J Clin Microbiol 61:e0174822. doi:10.1128/jcm.01748-22 36853028 PMC10035313

[22] Cartwright CP, Lembke BD, Ramachandran K, Body BA, Nye MB, Rivers CA, Schwebke JR. 2013. Comparison of nucleic acid amplification assays with BD affirm VPIII for diagnosis of vaginitis in symptomatic women. J Clin Microbiol 51:3694–3699. doi:10.1128/JCM.01537-13 23985917 PMC3889753

[23] Thompson A, Timm K, Borders N, Montoya L, Culbreath K. 2020. Diagnostic performance of two molecular assays for the detection of vaginitis in symptomatic women. Eur J Clin Microbiol Infect Dis 39:39–44. doi:10.1007/s10096-019-03694-w 31502121 PMC6962287

[24] Richter SS, Otiso J, Goje OJ, Vogel S, Aebly J, Keller G, Van Heule H, Wehn D, Stephens AL, Zanotti S, et al.. 2019. Prospective evaluation of molecular assays for diagnosis of vaginitis. J Clin Microbiol 58:e01264-19. doi:10.1128/JCM.01264-19 31694966 PMC6935898

[25] Dessai F, Nyirenda M, Sebitloane M, Abbai N. 2020. Diagnostic evaluation of the BD Affirm VPIII assay as a point-of-care test for the diagnosis of bacterial vaginosis, trichomoniasis and candidiasis. Int J STD AIDS 31:303–311. doi:10.1177/0956462419895684 32050856

[26] Andrea SB, Chapin KC. 2011. Comparison of Aptima *Trichomonas vaginalis* transcription-mediated amplification assay and BD affirm VPIII for detection of *T. vaginalis* in symptomatic women: performance parameters and epidemiological implications. J Clin Microbiol 49:866–869. doi:10.1128/JCM.02367-10 21248097 PMC3067695

[27] Nouioui I, Carro L, García-López M, Meier-Kolthoff JP, Woyke T, Kyrpides NC, Pukall R, Klenk HP, Goodfellow M, Göker M. 2018. Genome-based taxonomic classification of the phylum Actinobacteria Front Microbiol 9:2007. doi:10.3389/fmicb.2018.02007 30186281 PMC6113628

[28] Srinivasan S, Austin MN, Fiedler TL, Strenk SM, Agnew KJ, Gowda GAN, Raftery D, Beamer MA, Achilles SL, Wiesenfeld HC, et al.. 2023. *Amygdalobacter indicium* gen. nov., sp. nov., and *Amygdalobacter nucleatus* sp. nov., gen. nov.: novel bacteria from the family *Oscillospiraceae* isolated from the female genital tract. Int J Syst Evol Microbiol 73:006017. doi:10.1099/ijsem.0.006017 37787404 PMC11318147

[29] Srinivasan S, Beamer MA, Fiedler TL, Austin MN, Sizova MV, Strenk SM, Agnew KJ, Gowda GAN, Raftery D, Epstein SS, et al.. 2019. *Megasphaera lornae* sp. nov., *Megasphaera hutchinsoni* sp. nov., and *Megasphaera vaginalis* sp. nov.: novel bacteria isolated from the female genital tract. Int J Syst Evol Microbiol 71:004702. doi:10.1099/ijsem.0.004702 33616513 PMC8161547

[30] Sobel JD, Sobel R. 2018. Current treatment options for vulvovaginal candidiasis caused by azole-resistant *Candida* species. Expert Opin Pharmacother 19:971–977. doi:10.1080/14656566.2018.1476490 29932786

