# Supplementary material for: Comparing BD Affirm VPIII to Xpert Xpress MVP and BD MAX Vaginal Panel for the diagnosis of vaginal infections
Source: Microbiol Spectr. 2026 Jul 28;14(9):e02353-25. doi: 10.1128/spectrum.02353-25 (PMC13532205; doi:10.1128/spectrum.02353-25)
Supplement: Supplementary file 1 [file spectrum.02353-25-s0001.docx]

Supplementary Table 1. Sensitivity and Specificity of Affirm VPIII for Bacterial Vaginosis (BV), *Candida* species and *Trichomonas vaginalis*

|  | Either NAAT positive % | | | Both NAATs Positive % | | |
| --- | --- | --- | --- | --- | --- | --- |
|  |  |  |  |  |  |  |
| BV | Sensitivity: | 112/128 | 87.5 | Sensitivity: | 104/116 | 89.7 |
|  | Specificity: | 109/143 | 76.2 | Specificity: | 109/143 | 76.2 |
|  |  |  |  |  |  |  |
| *Candida* | Sensitivity: | 42/91 | 46.2 | Sensitivity: | 45/80 | 56.3 |
|  | Specificity: | 176/180 | 97.8 | Specificity: | 170/171 | 99.4 |
|  |  |  |  |  |  |  |
| *Trichomomas* | Sensitivity: | 6/14 | 42.9 | Sensitivity: | 6/14 | 42.9 |
|  | Specificity: | 257/257 | 100 | Specificity: | 257/257 | 100 |

| *Trichomomas* | Sensitivity: | 6/14 | 42.9 |
| --- | --- | --- | --- |
|  | Specificity: | 257/257 | 100 |
